# Supplementary material for: Base-edited CAR T cells for combinational therapy against T cell malignancies
Source: Leukemia. 2021 May 25;35(12):3466–81. doi: 10.1038/s41375-021-01282-6 (PMC8632682; doi:10.1038/s41375-021-01282-6)
Supplement: Supplementary file 1 — Supplemental Figures S1-S13 and tables S1-S4 with legends [file 41375_2021_1282_MOESM1_ESM.pdf]

Supplementary Figure 1

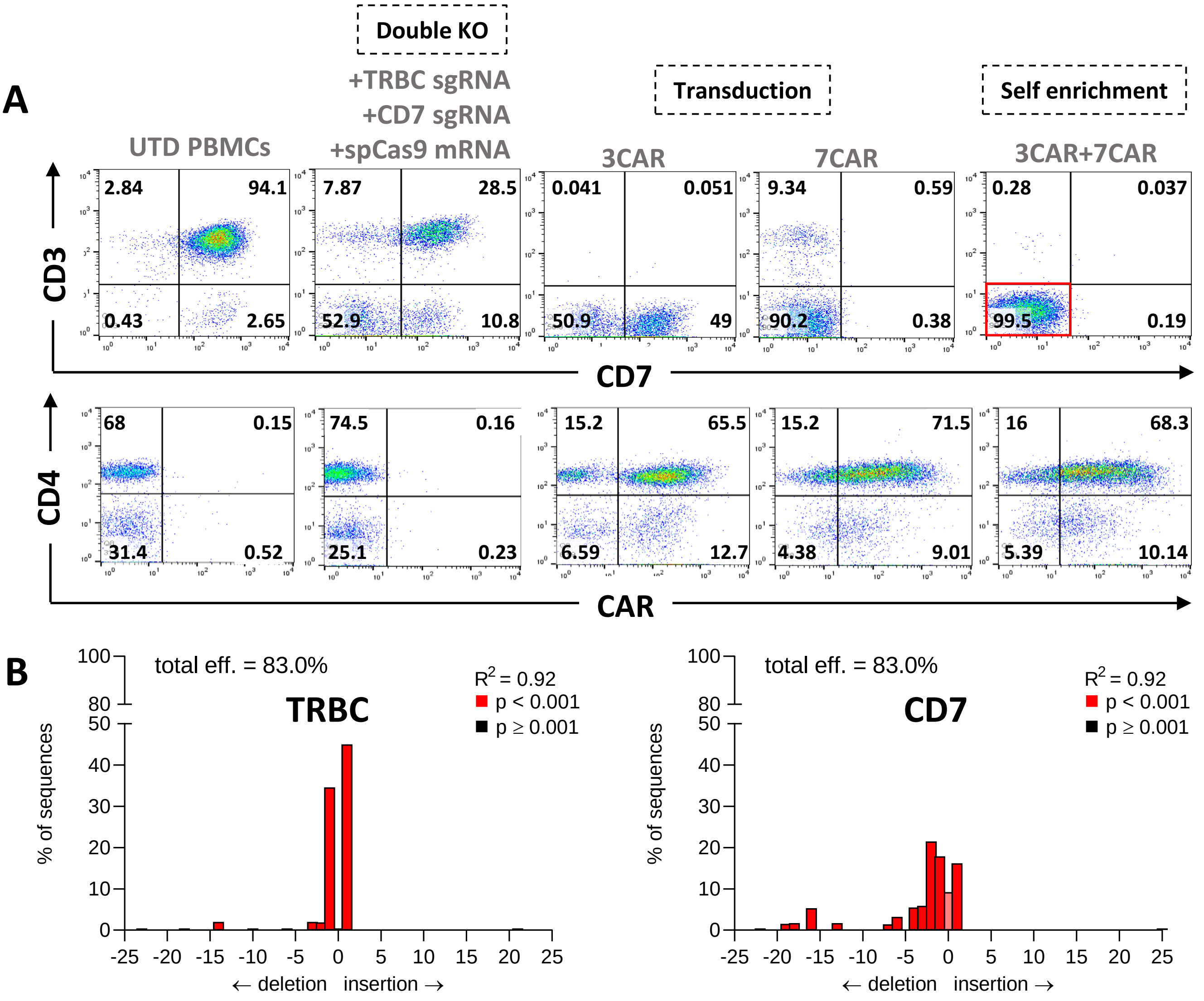

Supplementary Figure 1. SpCas9 3CAR and 7CAR primary T cells evade fratricide during production. **A)** Representative phenotypic analysis of CD3 and CD7 surface antigen expression (top panel) and CAR transduction (bottom panel) of CD3/CD28 activated PBMC cells elcetroporated with TRBC and CD7-targeting sgRNA and SpCas9 mRNA and subsequently transduced with 3CAR or 7CAR lentiviral vectors at MOI 5. Self-enrichment effects following co-culture of 3CAR and 7CAR products results in enriched $TCR^{-}CD7^{-}$ 3CAR/7CAR cells (red box). **B)** Representative TIDE output of Sanger sequencing results from mixed 3CAR/7CAR DNA PCR amplified using TRBC or CD7 forward and reverse primers. Percentage of target indels shown by red bars.

Supplementary Figure 2

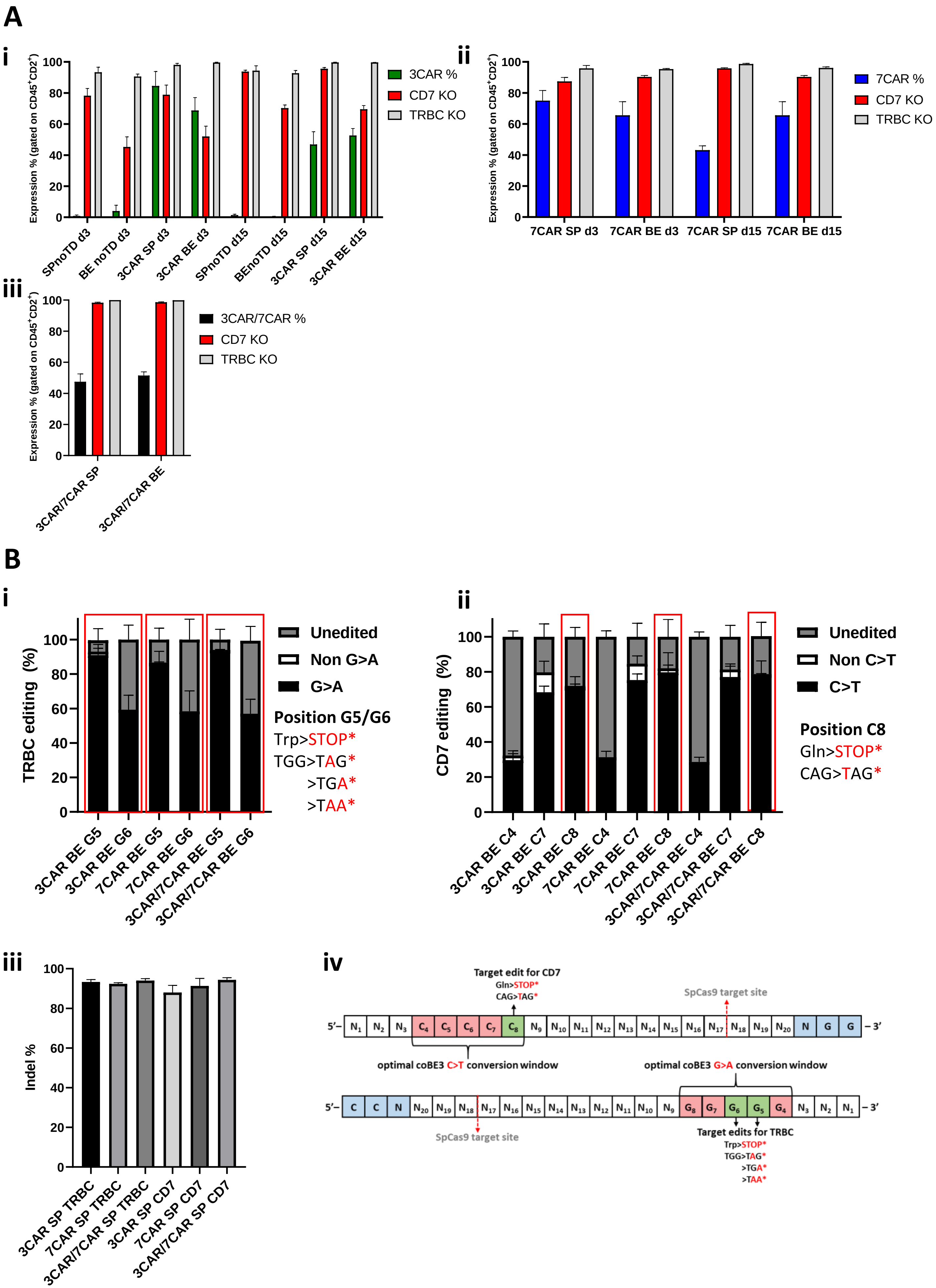

Supplementary Figure 2. **Comparison of phenotypic and molecular knockout of SpCas9 and coBE3 edited CAR T cells.**

A) T cells from healthy primary donors (n=3) were elcetroporated with either SpCas9 or coBE3 mRNA and sgRNAs targeting TRBC and CD7. Edited cells were transduced with either 3CAR **(i)**, or 7CAR **(ii)** lentiviral vectors before CAR expression and knockout were measured at early (d3) and late (d15) stage production. **(iii)** 3CAR and 7CAR effectors edited with either SpCas9 or coBE3 were co-cultured for 24 hours before undergoing similar phenotyping. **B)** DNA from these cells was extracted and the TRBC and CD7 loci were amplified by PCR for Sanger sequencing. **(i)** coBE3-mediated G>A or non G>A base conversion within the TRBC editing window, or **(ii)** C>T or non C>T base conversion within the TRBC editing window of CD7, were quantified by Editr software. **(iii)** SpCas9-mediated indels at TRBC or CD7 locus quantified by Synthego ICE analysis. Error bars represent SEM (n=3). **(iv)** Visual representation of sgRNA target sequence with rAPOBEC1 editing window highlighted. Target bases leading to premature STOP following coBE3-mediated editing highlighted in green. SpCas9 cleavage position marked by red arrow.

Supplementary Figure 3

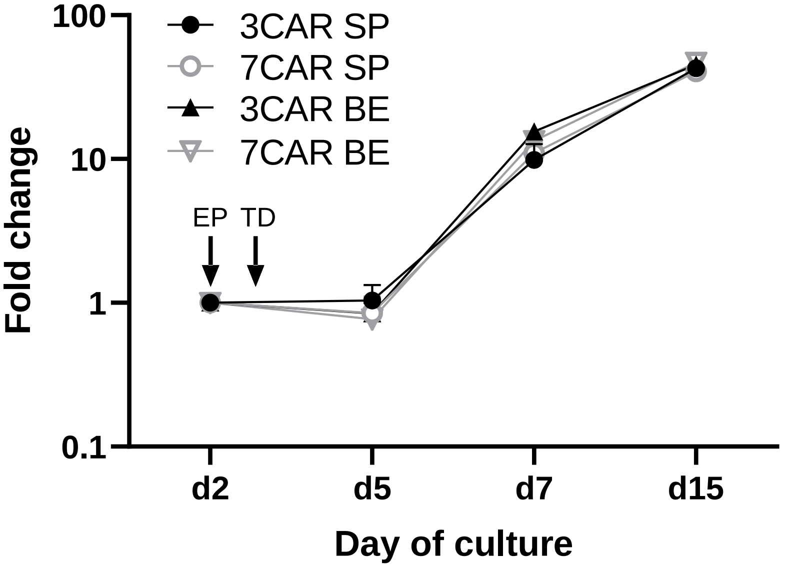

Supplementary Figure 3. coBE3-mediated removal of shared antigens on 3CAR and 7CAR T cells has no impact on cell expansion.

An equal starting number of cells from primary T cell donors (n=3) were activated on day 0 and then elcetroporated (EP) with either SpCas9 or coBE3 mRNA and sgRNAs targeting TRBC and CD7 on day 2 (d2). 24 hours later, cells were transduced (TD) with either 3CAR or 7CAR and cultured in G-Rex 24-well chambers. Cells were harvested and counted on days 5, 7 and 15 and fold expansion was measured and plotted. Error bars represent SEM across n=3 donors.

Supplementary Figure 4

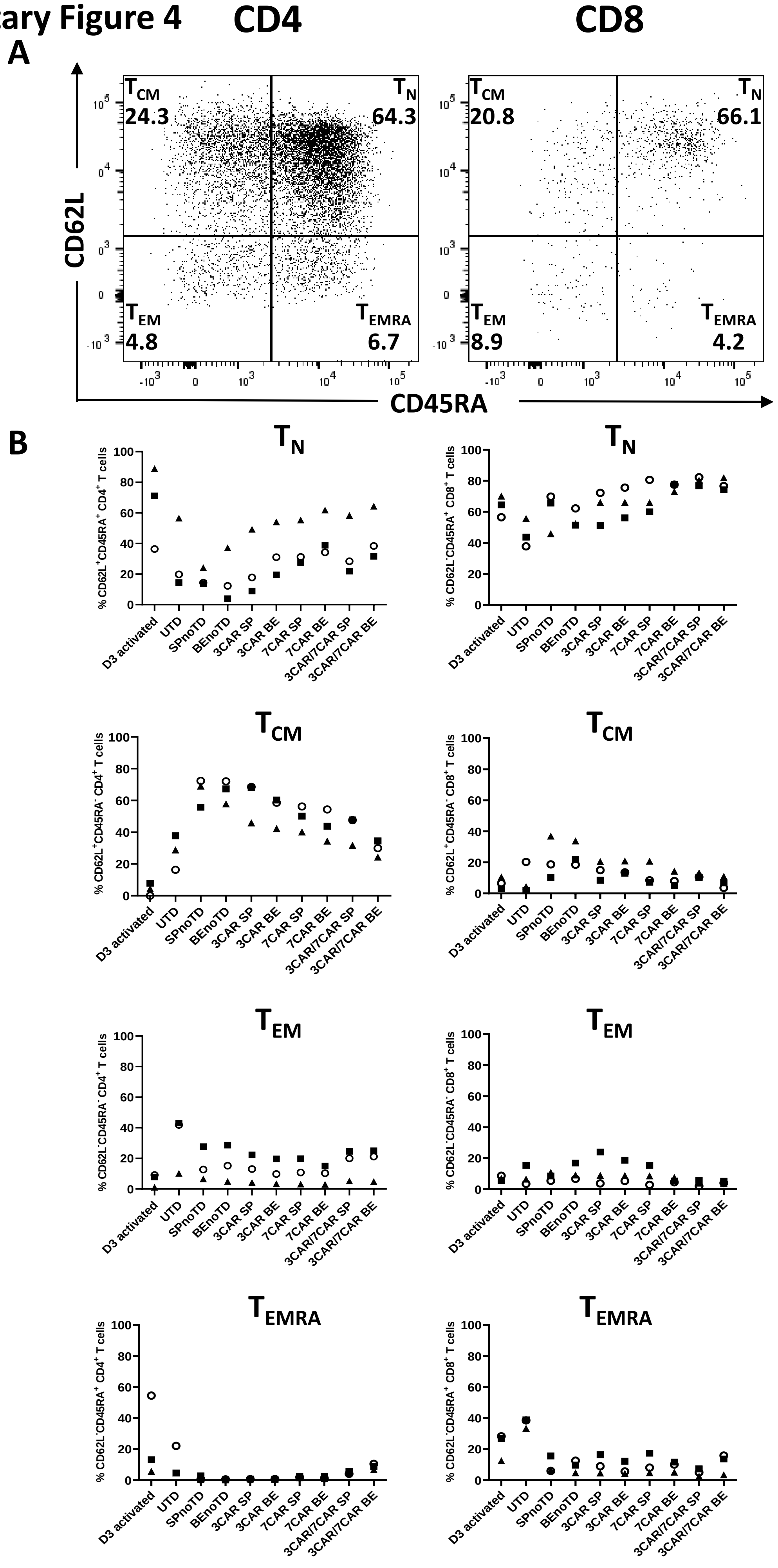

Supplementary Figure 4. Phenotype of 3CAR and 7CAR T cells with SpCas9 or coBE3 edits.

A) T cells from healthy primary donors (n=3) were elcetroporated with either SpCas9 or coBE3 mRNA and sgRNAs targeting TRBC and CD7. Edited cells were transduced with either 3CAR or 7CAR lentiviral vectors and cultured in G-Rex 24-well chambers prior to phenotyping. Representative flow cytometry plots from n=1 donor gated for $CD45^{+}CD2^{+}CD4^{+}$ (left) or $CD45^{+}CD2^{+}CD8^{+}$ (left) stained for CD62L and CD45RA allows for discrimination of CD62L+CD45RA+ naïve (TN), $CD62L^{+}CD45RA^{-}$ central memory (TCM), $CD62L^{-}CD45RA^{-}$ effector memory (TEM) or $CD62L^{-}CD45RA^{+}$ effector memory $CD45RA^{+}$ T cells (TEMRA). **B)** Differentiation status of T cells from primary donors (n=3) individually depicted as open circles, squares or triangles, according to CD4, CD8, CD62L and CD45RA expression.

Supplementary Figure 5

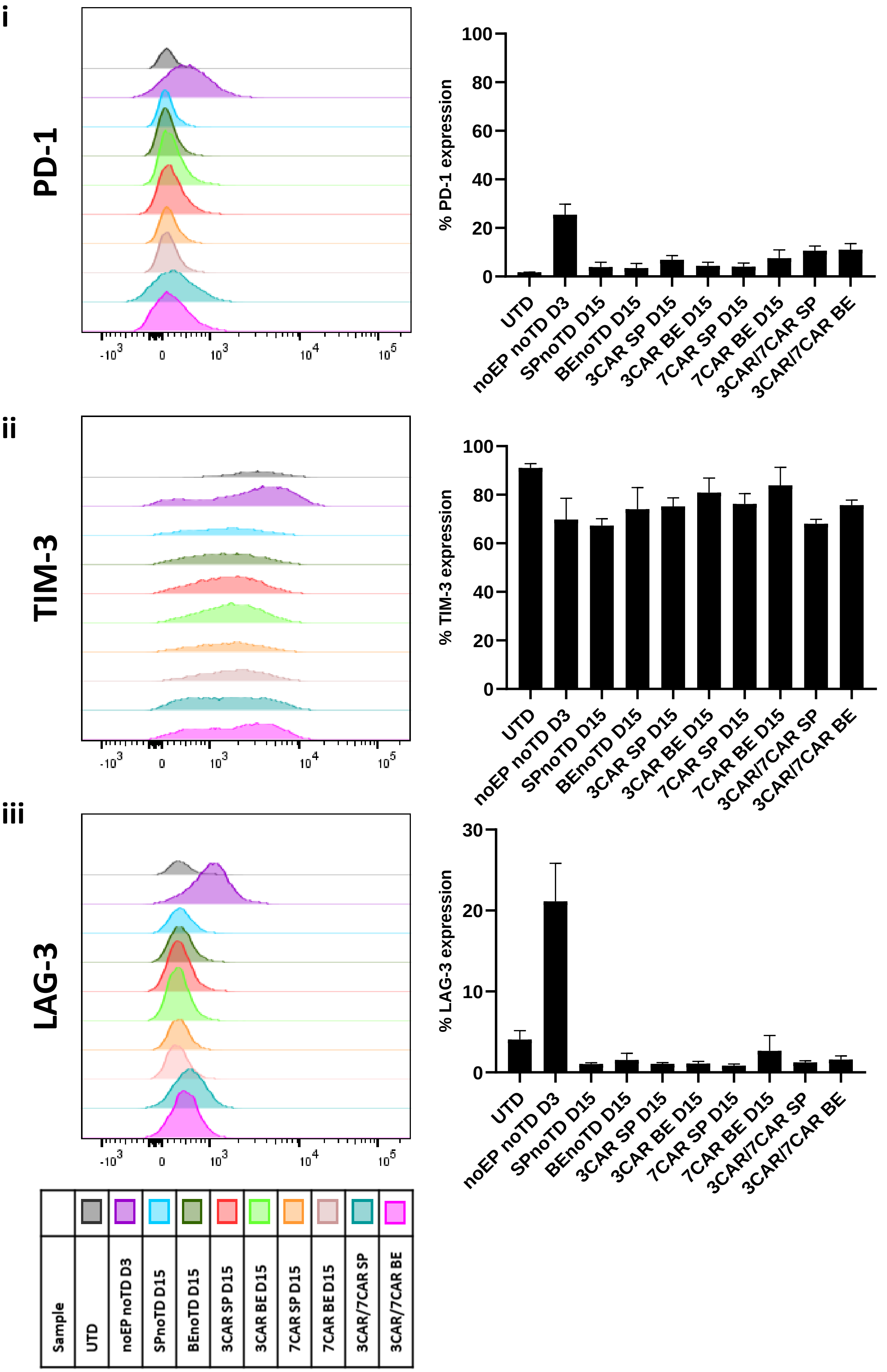

Supplementary Figure 5. Exhaustion marker expression following SpCas9 editing or cytidine deamination in 3CAR and 7CAR T cells.

T cells from n=3 healthy primary donors were elcetroporated with either SpCas9 or coBE3 mRNA and sgRNAs targeting TRBC and CD7. Edited cells were transduced with either 3CAR or 7CAR lentiviral vectors and cultures for 15 days. Cells (gated on $CD45^{+}CD2^{+}$) were analysed for expression of **(i)** PD-1, **(ii)** TIM-3 and **(iii)** LAG-3 at end of culture. Panels on the left depicts representative mean fluorescence intensity (MFI) data from n=1 of 3 donors. Histograms on the right quantify expression of each marker across n=3 donors. Error bars represent SEM across n=3 donors.

Supplementary Figure 6

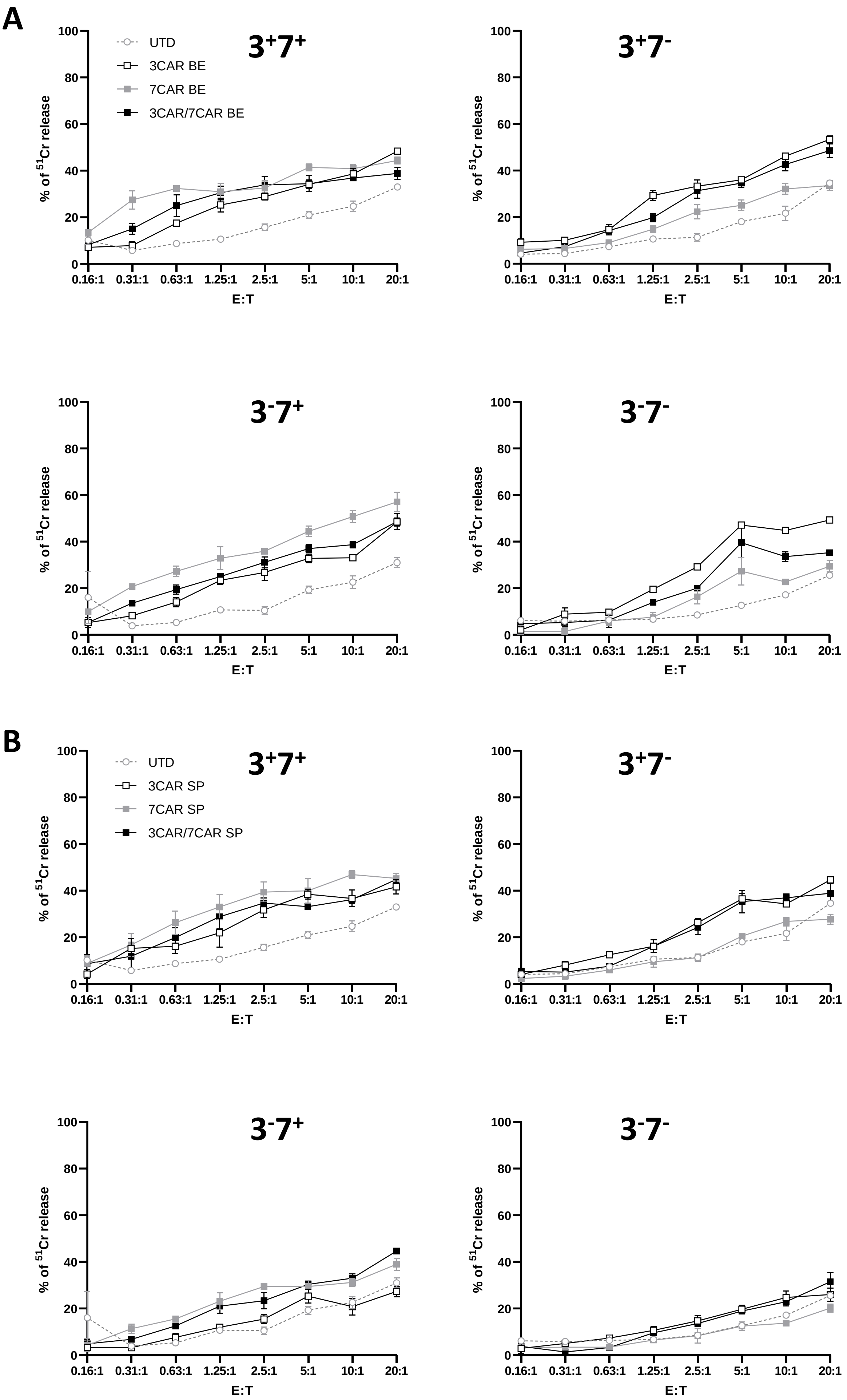

Supplementary Figure 6. 3CAR/7CAR T cells mediate potent killing of T-ALL cells in vitro.

In vitro cytotoxicity of 3CAR and 7CAR cells against T-ALL cell line targets. **A)** ^{51}Cr labelled Jurkat T cells modified to express $CD3^{+}CD7^{+}$, $CD3^{+}CD7^{-}$, $CD3^{-}CD7^{+}$ or $CD3^{-}CD7^{-}$ were co-cultured with coBE3 edited $CD3^{-}CD7^{-}$ 3CAR (white squares), 7CAR (grey squares), mixed 3CAR/7CAR (black squares) or untransduced (white circles) cells at an increasing ratio of effectors:targets (E:T). **B)** ^{51}Cr labelled Jurkat T cell interactions co-cultured with SpCas9 edited $CD3^{-}CD7^{-}$ 3CAR (white squares), 7CAR (grey squares), mixed 3CAR/7CAR (black squares) or untransduced (white circles) cells at an increasing ratio of effectors:targets (E:T). Error bars represent SEM of (n=3) technical replicates.

Supplementary Figure 7

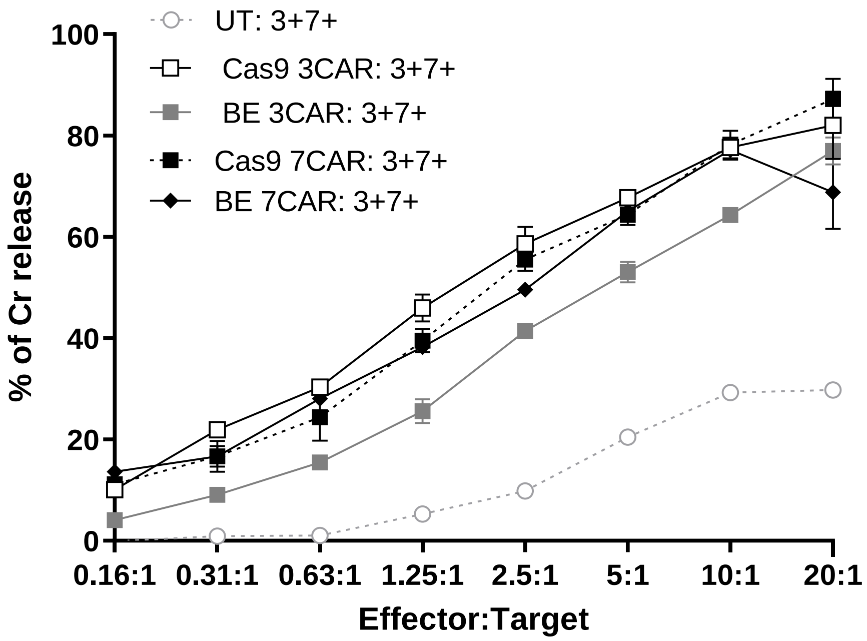

Supplementary Figure 7. SpCas9 and coBE3 3CAR and 7CAR primary T cells demonstrate comparable target cell lysis *in vitro.*

In vitro cytotoxic activity of SpCas9 or coBE3 edited 3CAR, 7CAR cells or untransduced negative control effector T cells against ^{51}Cr loaded $CD3^{+}CD7^{+}$ Jurkat T cell targets at an increasing effector:target (E:T) ratio. Points represent mean of n=3 experimental triplicates and error bars represent SEM.

Supplementary Figure 8

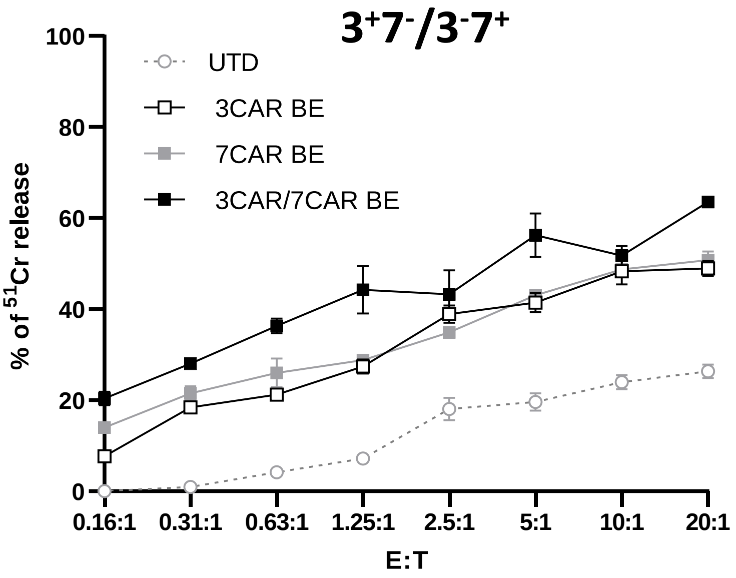

Supplementary Figure 8. Combined 3CAR/7CAR T cells exhibit efficient lysis of heterogeneous $CD3^{+/-}$ $CD7^{+/-}$ T-ALL cells in vitro.

In vitro cytotoxicity of base edited 3CAR and 7CAR cells against mixed $CD3^{+/-}CD7^{+/-}$ T-ALL cell line targets. ^{51}Cr labelled $CD3^{+}CD7^{-}$, $CD3^{-}CD7^{+}$ modified Jurkat T cells mixed at a ratio of 1:1 were co-cultured with coBE3 edited $CD3^{-}CD7^{-}$ 3CAR (white squares), 7CAR (grey squares), mixed 3CAR/7CAR (black squares) or untransduced (white circles) cells at an increasing ratio of effectors:targets (E:T). Error bars represent SEM of (n=3) technical replicates.

Supplementary Figure 9

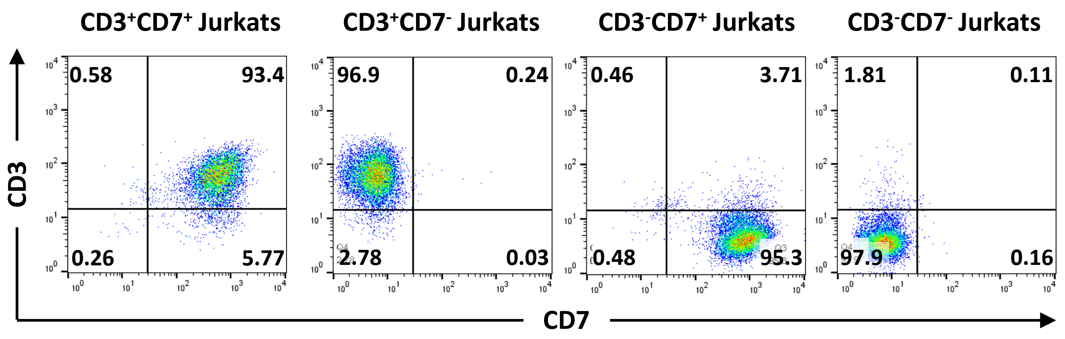

Supplementary Figure 9. Jurkat T cells modified for to express CD3 and/or CD7 surface antigen.

Jurkat T cells electroporated with SpCas9 mRNA and TRBC and/or CD7 targeting sgRNAs and sorted for $CD3^{+}CD7^{+}$, $CD3^{+}CD7^{-}$, $CD3^{-}CD7^{+}$ and $CD3^{-}CD7^{-}$. Flow cytometry of CD3 and CD7 expression of edited and sorted Jurkat T cells confirming surface antigen expression.

Supplementary Figure 10

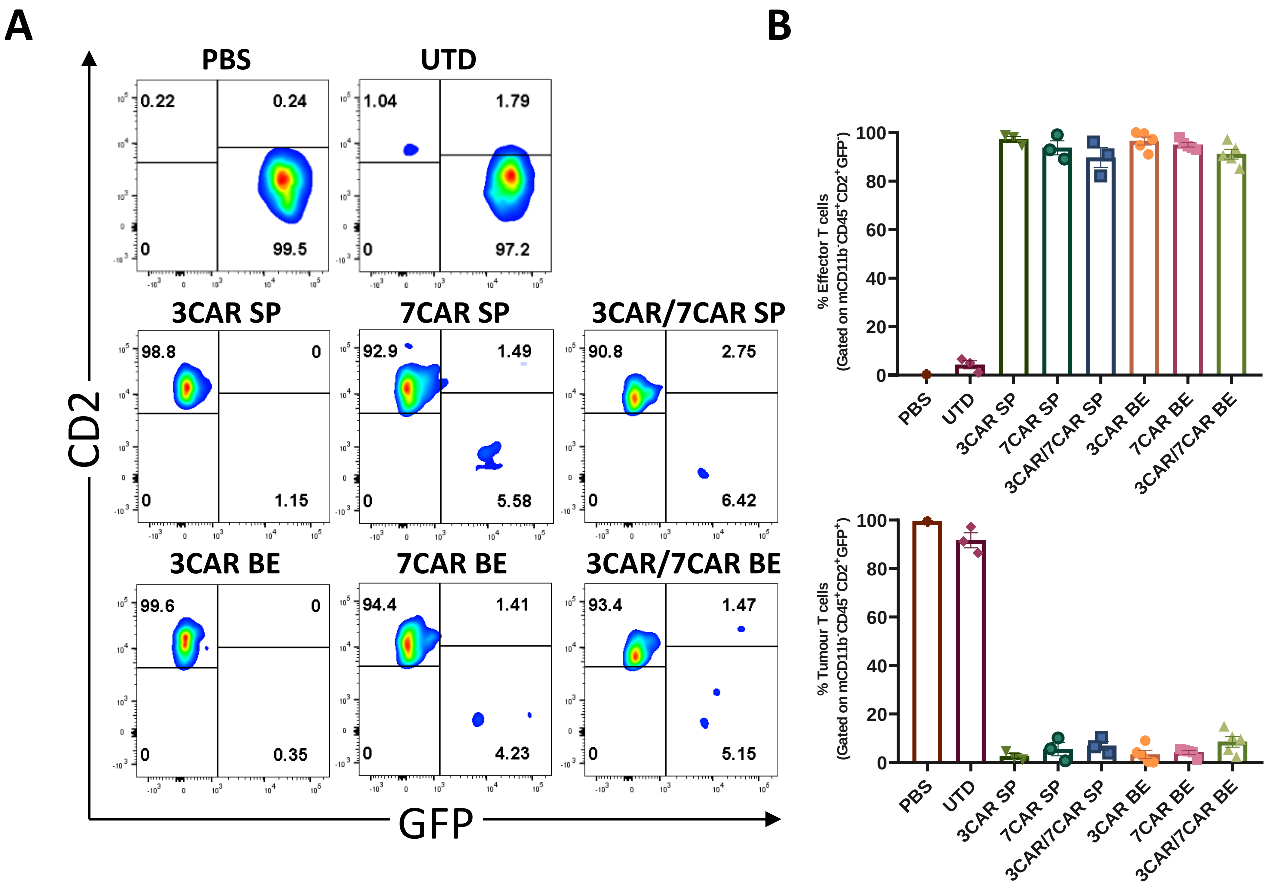

Supplementary Figure 10. Base edited 3CAR and 7CAR cells demonstrate comparable anti-tumour clearance in vivo to CRISPR-Cas9 edited cells.

NOD/SCID/$\gamma c^{-/-}$ (NSG) mice (n=28) were intravenously injected with 10 x 10^6 GFP^+LUC^+ $CD3^+CD7^+$ Jurkat T cells prior to receiving an IV infusion of $10x10^6$ SpCas9 edited TCR^-CD7^- 3CAR (n=3), $10x10^6$ SpCas9 edited TCR^-CD7^- 7CAR (n=3), $10x10^6$ SpCas9 edited TCR^-CD7^- 3CAR/7CAR (n=3), $10x10^6$ coBE3 edited TCR^-CD7^- 3CAR (n=5), $10x10^6$ coBE3 edited TCR^-CD7^- 7CAR (n=5), or $10x10^6$ coBE3 edited TCR^-CD7^- 3CAR/7CAR (n=5) effectors. Control mice received untransduced (UTD) cells (n=3) or PBS (n=1). Leukemic progression was monitored by serial bioluminescent imaging (BLI) until day 31 when all groups were sacrificed. **A)** Representative flow cytometry plots of effector ($CD2^+GFP^-$) and tumour ($CD2^+GFP^+$) subpopulations detected in bone marrow aspirates subgated on Live>mCD11b->$hCD45^+$. **B)** Proportion of effector T cells (top) or tumour cells (bottom) when subgated on Live>mCD11b->$hCD45^+$. Error bars represent SEM. ***$P< 0.001$ ****$P< 0.0001$.

Supplementary Figure 11

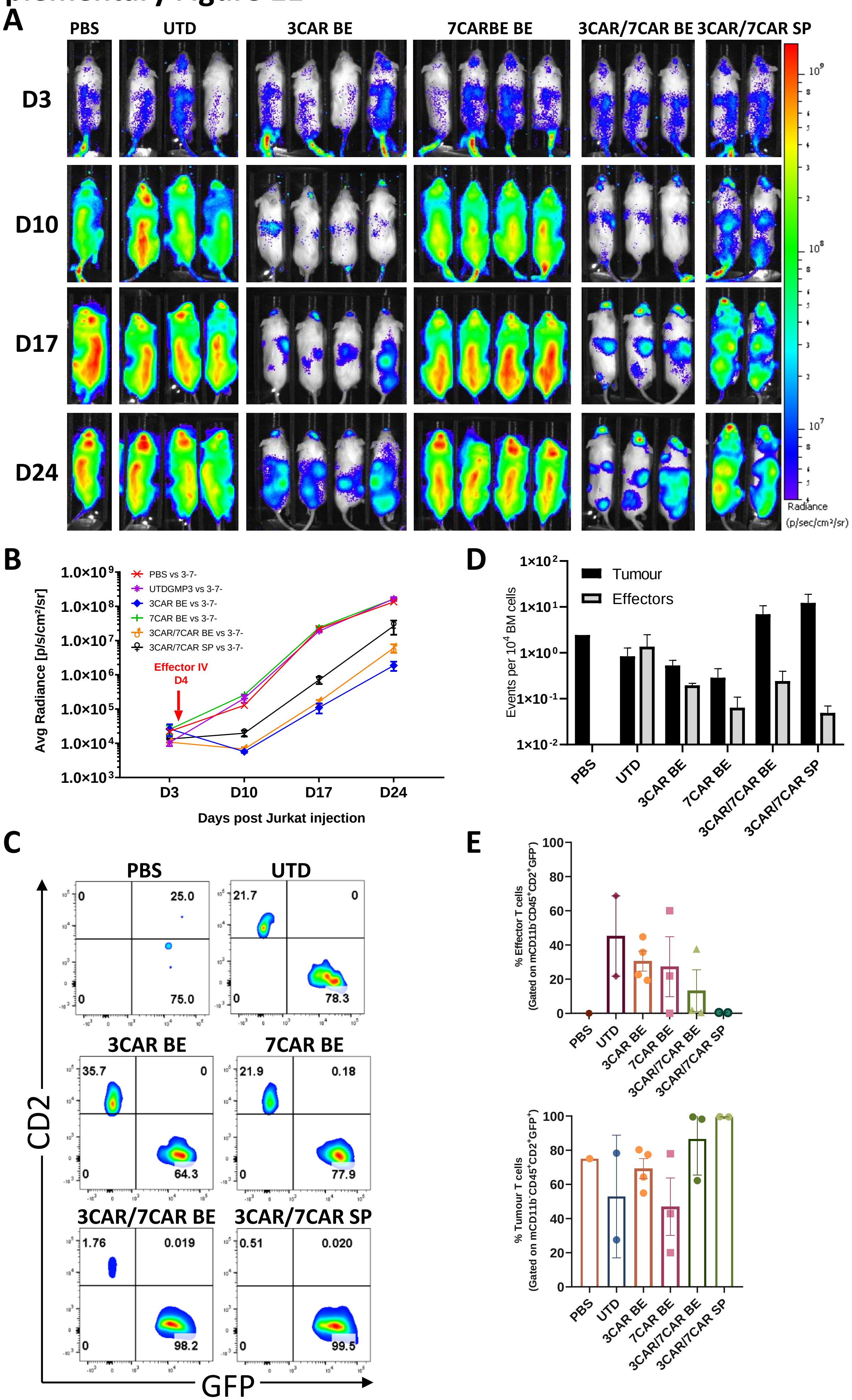

Supplementary Figure 11. Target CD3 and CD7 antigen ablation leads to tumour progression in in vivo model.

A) n=17 NOD/SCID/$\gamma c^{-/-}$ (NSG) mice were intravenously injected with 10 x 10^6 GFP^+LUC^+ Jurkat T cells edited for the disruption of CD3 and CD7 (Day 0) prior to receiving an IV infusion of effector T cells (Day 4). Mice received either 10x10^6 coBE3 edited TCR^-CD7^- 3CAR (n=4), 10x10^6 coBE3 edited TCR^-CD7^- 7CAR (n=4), 10x10^6 coBE3 edited TCR^-CD7^- 3CAR/7CAR (n=3), or 10x10^6 SpCas9 edited TCR^-CD7^- 3CAR/7CAR (n=2) effectors. Control mice received untransduced (UTD) cells from an unrelated donor (n=3) or PBS (n=1). Leukemic progression was monitored by serial bioluminescent imaging (BLI) until day 24 when all groups were sacrificed. **B)** Bioluminescence signal of each animal plotted as Average radiance [photons/sec/cm^2/sr]. Each line represents a different experimental group and each point on the line the mean of each group. Error bars represent SEM. **C)** Representative flow cytometry plots of effector ($CD2^+GFP^-$) and tumour ($CD2^+GFP^+$) subpopulations detected in bone marrow aspirates subgated on Live>mCD11b->$hCD45^+$. **D)** Number of effector events or tumour events per 1x10^4 acquired bone marrow events. **E)** Proportion of effector T cells (top) or tumour cells (bottom) when subgated on Live>mCD11b->$hCD45^+$. Error bars represent SEM.

Supplementary Figure 12

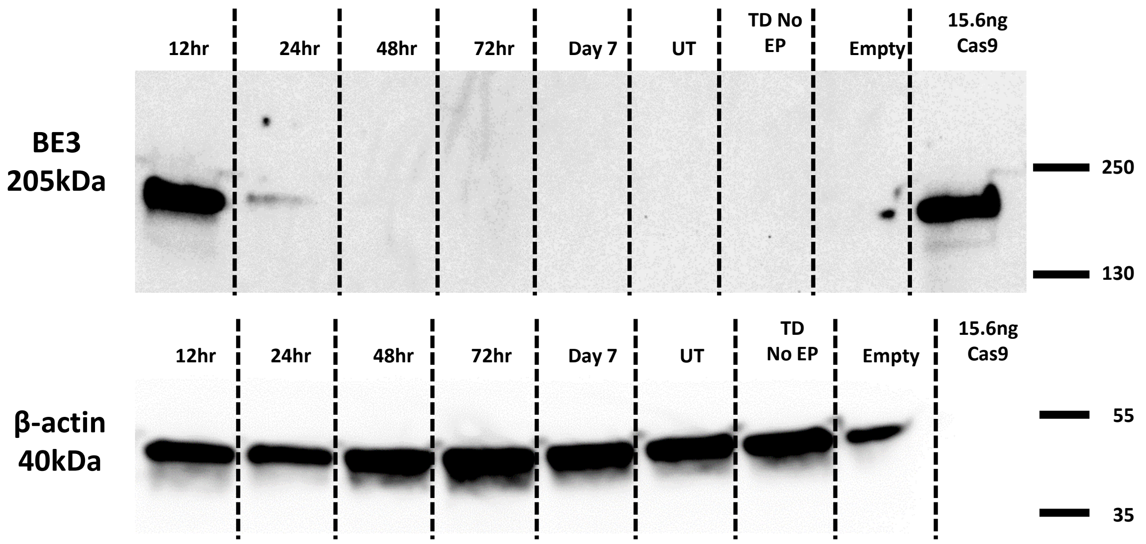

Supplementary Figure 12. Base editor protein dilution following primary T cell culture.

Cell lysates from primary T cells transduced with a Terminal-TRAC-CAR19 lentiviral vector at MOI 5 and exposed to coBE3 mRNA (50µg/ml) by electroporation were collected 12, 24, 48, 72 hours post culture and again on day 7 of culture. Untransduced cell lysate was also collected at day 7 and used as a negative control. 20µg of BCA-normalised total protein was loaded on a 4%-15% SDS-PAGE alongside positive SpCas9 protein control (15.6ng). Membrane was probed with anti-CRISPR-cas9 primary and sheep anti-mouse-HRP secondary antibody. Cas9 positive bands detected at 205kDa. Membrane was probed with anti b-actin primary antibody and sheep anti-mouse-HRP secondary antibody as a marker of loading detected at 40 kDa.

Supplementary Figure 13

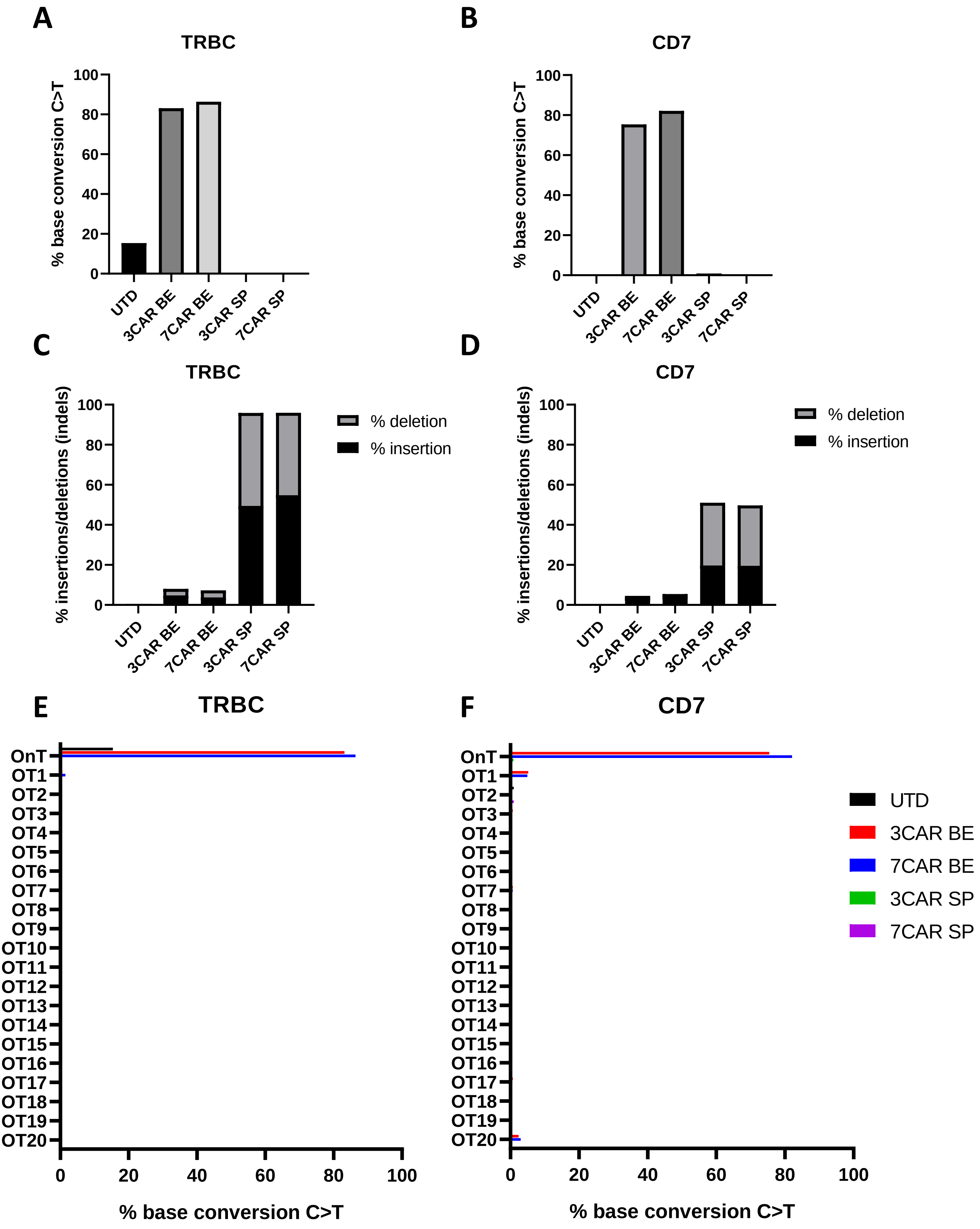

Supplementary Figure 13. Identification of base conversion and indel frequencies at *in silico* predicted 'off-target' sites.

DNA from T cells edited at the TRBC and CD7 loci with either coBE3 or SpCas9 mRNA and transduced with 3CAR or 7CAR was subjected to next generation sequencing for the detection of on-target (OnT) NHEJ events or C>T base conversion across the top 20 guide dependent *in silico* predicted 'off-target' (OT) sites for TRBC and CD7 targeting sgRNAs. On-target C>T base conversion frequency in coBE3 or SpCas9 edited samples at **A)** TRBC, and **B)** CD7 loci. Insertion or deletion frequency leading to NHEJ in coBE3 or SpCas9 edited samples at on-target **C)** TRBC, and **D)** CD7 genomic sites. C>T base conversion at top 20 'off-target' sites for **E)** TRBC, and **F)** CD7 presented as average conversion across C bases within the 5 bp editing window of rAPOBEC1.

Supplementary Table S1. Target-specific primer combinations for NGS on top 20 Benchling off-targets for TRBC and CD7 target sequences. Following an internally-built workflow, a 20bp window around the predicted off-target nucleotide position, was analysed to a minor allele frequency of 0.01%. Primer sequences are given in the 5'-3' orientation. Fwd: forward, Rev: reverse.

Off-target	Sequence	PAM	Chromosome	ChromStart	ChromEnd
TRBC-on	CCCACCAGCTCAGCTCCACG	TGG	chr7	142498843	142498862
TRBC-01	CCTTCCAGCTCAGCTCCACG	TAG	chr19	47660965	47660984
TRBC-02	TCCACCACGTCAGCTCCACG	GAG	chr9	127039147	127039166
TRBC-03	CTCACCAAGTCAGCTCCACG	CAG	chr20	31603858	31603877
TRBC-04	GCCACAATCTCAGCTCCACG	GGG	chr6	151908255	151908274
TRBC-05	CTCACCAGCCAAGCTCCACG	GGG	chr5	152916938	152916957
TRBC-06	CCCAGCAGTCCAGCTCCACG	CAG	chr17	43195462	43195481
TRBC-07	GCCATCAGCTCAGCTCCATG	CAG	chr6	51981591	51981610
TRBC-08	GCCAGCAGCTCAGCTCCATG	TGG	chr2	240938429	240938448
TRBC-09	ATCACCAGCTCAGCTCCAGG	CGG	chr9	87606587	87606606
TRBC-10	CTGAGCAGCACAGCTCCACG	CAG	chr4	186761189	186761208
TRBC-11	CAGGCCAGCCCAGCTCCACG	AGG	chr1	201390814	201390833
TRBC-12	CCCCACACCCCAGCTCCACG	AGG	chr17	79561515	79561534
TRBC-13	CCGTCCAGCTCAGCTCCAAG	GGG	chr17	71281007	71281026
TRBC-14	CCGACCACCTCAGCTCCAGG	AAG	chr21	26458611	26458630
TRBC-15	GCCACCCGCTCAGCTCCAAG	CAG	chrX	47297557	47297576
TRBC-16	ACCATCATGTCAGCTCCACG	AGG	chr22	50925765	50925784
TRBC-17	GACACCAACTCAGCTCCACT	CAG	chr11	70677520	70677539
TRBC-18	CACCACAGTTCAGCTCCACG	GAG	chr16	20071470	20071489
TRBC-19	CCCACCCACTCGGCTCCACG	CAG	chr16	89055350	89055369
TRBC-20	TGCATAAGCTCAGCTCCACG	CAG	chr8	40188393	40188412

Off-target	Sequence	PAM	Chromosome	ChromStart	ChromEnd
CD7-on	CACCTGCCAGGCCATCACGG	AGG	chr17	80274585	80274604
CD7-01	CTCCTGCCAGGCCATCATGG	GGG	chr17	16384936	16384955
CD7-02	CACCAGCGAGACCATCACGG	CGG	chr5	168347501	168347520
CD7-03	CCCCGGCCAGGCCATCACAG	GGG	chr17	77615816	77615835
CD7-04	CCCCGGCCAGGCCATCACAG	GGG	chr17	77514495	77514514
CD7-05	CACCTGCCAGGGCATCACAG	GAG	chr17	38518298	38518317
CD7-06	CGCCAGCATGGCCATCACGG	GAG	chr6	4511412	4511431
CD7-07	CAGCCTCAAGGCCATCACGG	CGG	chr9	131584856	131584875
CD7-08	CAACTGCCAGACCATCACTG	CAG	chr8	128695088	128695107
CD7-09	TCCCTGCAAGGCCATCACTG	TGG	chr5	5768818	5768837
CD7-10	CACCTGCCAGGCCATCTCTG	TGG	chr10	134652817	134652836
CD7-11	CTTCTTCCAGCCCATCACGG	AGG	chr17	712317	712336
CD7-12	CTCCTGCCAGCCCAGCACGG	CAG	chr5	7662631	7662650
CD7-13	CTCCGGACAGGCCATCTCGG	GGG	chr1	17511832	17511851
CD7-14	CCACTTCCAGGGCATCACGG	GAG	chr10	99628963	99628982
CD7-15	ATCCTGGCAGGCCATCACTG	AAG	chr18	42523708	42523727
CD7-16	AGCCTGTCAGGCCATCACAG	AAG	chr20	52447203	52447222
CD7-17	CAGCCTCCAGGACATCACGG	AGG	chrX	591794	591813
CD7-18	CTCCTGCCAGCCCATCAGGG	TAG	chr19	19215793	19215812
CD7-19	GCCCTGCCTGGCCATCACCG	CAG	chr9	100158442	100158461
CD7-20	TCCCTCCCAGGCCATCACTG	AGG	chr12	58303936	58303955

Supplementary Table S2: Next generation sequencing data analysis of top 20 guide-dependent off-target sites for **A)** TRBC or **B)** CD7. Percentage C>T conversion in coBE3 or SpCas9 treated samples across C bases within each 20 bp off-target sequence.

A)

Gene	OT	C position	% base conversion C>T				
			UTD	3CAR BE	7CAR BE	3CAR SP	7CAR SP
TRBC	1	19	0.00	0.02	0.05	0.03	0.02
TRBC	1	17	0.01	0.02	0.05	0.01	0.00
TRBC	1	16	0.04	0.02	0.02	0.01	0.00
TRBC	1	14	0.02	0.04	0.05	0.01	0.00
TRBC	1	11	0.03	0.02	0.36	0.04	0.02
TRBC	1	9	0.03	0.09	0.02	0.00	0.02
TRBC	1	6	0.02	0.49	1.34	0.00	0.00
TRBC	1	5	0.02	0.67	1.55	0.05	0.00
TRBC	1	2	0.03	0.02	0.10	0.04	0.10
TRBC	1	1	0.02	0.04	0.06	0.00	0.00
TRBC	2	19	0.00	0.00	0.09	0.00	0.00
TRBC	2	17	0.06	0.00	0.00	0.00	0.00
TRBC	2	16	0.00	0.00	0.00	0.09	0.00
TRBC	2	14	0.06	0.00	0.08	0.00	0.00
TRBC	2	11	0.00	0.00	0.00	0.00	0.00
TRBC	2	8	0.00	0.00	0.17	0.00	0.00
TRBC	2	6	0.12	0.12	0.08	0.09	0.00
TRBC	2	5	0.00	0.00	0.08	0.00	0.00
TRBC	2	3	0.00	0.00	0.08	0.00	0.00
TRBC	2	2	0.00	0.12	0.16	0.00	0.00
TRBC	3	19	0.00	0.00	0.26	0.00	0.07
TRBC	3	17	0.00	0.00	0.00	0.00	0.04
TRBC	3	16	0.00	0.00	0.00	0.04	0.07
TRBC	3	14	0.00	0.00	0.00	0.00	0.04
TRBC	3	11	0.10	0.00	0.00	0.00	0.00
TRBC	3	6	0.00	0.00	0.00	0.00	0.04
TRBC	3	5	0.05	0.00	0.00	0.08	0.00
TRBC	3	3	0.00	0.00	0.00	0.04	0.00
TRBC	3	1	0.00	0.00	0.00	0.00	0.00
TRBC	4	19	0.04	0.15	0.00	0.05	0.01
TRBC	4	17	0.04	0.06	0.00	0.06	0.04
TRBC	4	16	0.08	0.00	0.07	0.00	0.04
TRBC	4	14	0.04	0.09	0.00	0.02	0.01
TRBC	4	11	0.00	0.09	0.07	0.00	0.06
TRBC	4	9	0.00	0.12	0.00	0.02	0.01
TRBC	4	5	0.00	0.06	0.13	0.02	0.03
TRBC	4	3	0.04	0.06	0.07	0.05	0.10
TRBC	4	2	0.00	0.00	0.07	0.02	0.01
TRBC	5	19	0.03	0.05	0.12	0.01	0.05
TRBC	5	17	0.06	0.05	0.11	0.02	0.07

TRBC	5	16	0.00	0.09	0.04	0.01	0.03
TRBC	5	14	0.03	0.12	0.05	0.06	0.04
TRBC	5	10	0.06	0.05	0.14	0.08	0.03
TRBC	5	9	0.01	0.04	0.00	0.04	0.03
TRBC	5	6	0.01	0.09	0.11	0.06	0.03
TRBC	5	5	0.06	0.05	0.13	0.03	0.05
TRBC	5	3	0.01	0.05	0.04	0.02	0.03
TRBC	5	1	0.06	0.11	0.08	0.09	0.06
TRBC	6	1	0.10	0.00	0.00	0.00	0.00
TRBC	6	2	0.05	0.08	0.00	0.00	0.04
TRBC	6	3	0.05	0.24	0.07	0.00	0.08
TRBC	6	6	0.05	0.00	0.07	0.00	0.04
TRBC	6	10	0.10	0.08	0.07	0.05	0.08
TRBC	6	11	0.05	0.15	0.00	0.00	0.04
TRBC	6	14	0.05	0.07	0.07	0.05	0.04
TRBC	6	16	0.14	0.15	0.07	0.00	0.04
TRBC	6	17	0.10	0.08	0.00	0.05	0.04
TRBC	6	19	0.00	0.08	0.00	0.00	0.12
TRBC	7	2	0.05	0.09	0.05	0.04	0.05
TRBC	7	3	0.07	0.03	0.03	0.06	0.06
TRBC	7	6	0.02	0.01	0.02	0.02	0.02
TRBC	7	9	0.05	0.03	0.03	0.05	0.02
TRBC	7	11	0.05	0.03	0.04	0.08	0.04
TRBC	7	14	0.04	0.02	0.03	0.05	0.06
TRBC	7	16	0.05	0.01	0.01	0.02	0.05
TRBC	7	17	0.05	0.05	0.06	0.05	0.05
TRBC	8	17	0.06	0.10	0.09	0.10	0.07
TRBC	8	16	0.06	0.07	0.06	0.07	0.08
TRBC	8	14	0.05	0.12	0.06	0.09	0.03
TRBC	8	11	0.06	0.01	0.07	0.08	0.05
TRBC	8	9	0.03	0.10	0.07	0.08	0.05
TRBC	8	6	0.05	0.12	0.06	0.10	0.03
TRBC	8	3	0.07	0.03	0.07	0.07	0.07
TRBC	8	2	0.09	0.03	0.09	0.09	0.02
TRBC	9	17	0.01	0.02	0.02	0.05	0.03
TRBC	9	16	0.01	0.02	0.02	0.03	0.02
TRBC	9	14	0.02	0.02	0.02	0.01	0.08
TRBC	9	11	0.01	0.02	0.02	0.02	0.17
TRBC	9	9	0.01	0.01	0.02	0.00	0.05
TRBC	9	6	0.03	0.02	0.05	0.03	0.63
TRBC	9	5	0.01	0.03	0.02	0.04	0.31
TRBC	9	3	0.02	0.02	0.07	0.02	0.50
TRBC	10	1	0.02	0.03	0.01	0.01	0.02
TRBC	10	6	0.05	0.04	0.03	0.03	0.02
TRBC	10	9	0.03	0.01	0.03	0.04	0.04
TRBC	10	11	0.02	0.00	0.01	0.02	0.04
TRBC	10	14	0.02	0.01	0.02	0.02	0.04

TRBC	10	16	0.02	0.06	0.02	0.03	0.05
TRBC	10	17	0.02	0.03	0.03	0.03	0.04
TRBC	10	19	0.03	0.03	0.04	0.03	0.08
TRBC	11	19	0.04	0.02	0.00	0.03	0.05
TRBC	11	17	0.00	0.00	0.00	0.03	0.02
TRBC	11	16	0.08	0.05	0.00	0.09	0.02
TRBC	11	14	0.02	0.03	0.04	0.10	0.00
TRBC	11	11	0.02	0.03	0.02	0.03	0.10
TRBC	11	10	0.00	0.09	0.10	0.03	0.05
TRBC	11	9	0.12	0.09	0.00	0.06	0.00
TRBC	11	6	0.06	0.12	0.08	0.06	0.00
TRBC	11	5	0.06	0.02	0.04	0.03	0.05
TRBC	11	1	0.02	0.00	0.00	0.00	0.05
TRBC	12	1	0.01	0.02	0.02	0.04	0.03
TRBC	12	2	0.02	0.03	0.02	0.02	0.02
TRBC	12	3	0.03	0.02	0.03	0.02	0.04
TRBC	12	4	0.03	0.03	0.01	0.02	0.02
TRBC	12	6	0.36	0.33	0.29	0.26	0.28
TRBC	12	8	0.03	0.02	0.01	0.02	0.02
TRBC	12	9	0.03	0.02	0.01	0.02	0.03
TRBC	12	10	0.02	0.05	0.02	0.06	0.04
TRBC	12	11	0.30	0.20	0.21	0.17	0.19
TRBC	12	14	0.30	0.18	0.19	0.16	0.16
TRBC	12	16	0.02	0.03	0.02	0.03	0.01
TRBC	12	17	0.03	0.07	0.05	0.02	0.05
TRBC	12	19	0.04	0.08	0.08	0.04	0.05
TRBC	13	1	0.05	0.07	0.02	0.07	0.07
TRBC	13	2	0.07	0.14	0.10	0.03	0.21
TRBC	13	5	0.05	0.04	0.08	0.03	0.03
TRBC	13	6	0.05	0.04	0.06	0.03	0.03
TRBC	13	9	0.00	0.04	0.02	0.00	0.03
TRBC	13	11	0.02	0.00	0.06	0.07	0.00
TRBC	13	14	0.05	0.04	0.02	0.00	0.00
TRBC	13	16	0.08	0.00	0.09	0.04	0.00
TRBC	13	17	0.05	0.08	0.07	0.00	0.04
TRBC	14	1	0.09	0.03	0.10	0.11	0.18
TRBC	14	2	0.05	0.06	0.09	0.10	0.05
TRBC	14	5	0.00	0.00	0.08	0.03	0.04
TRBC	14	6	0.02	0.03	0.02	0.00	0.02
TRBC	14	8	0.00	0.07	0.04	0.00	0.00
TRBC	14	9	0.00	0.01	0.06	0.00	0.03
TRBC	14	11	0.07	0.01	0.00	0.02	0.09
TRBC	14	14	0.00	0.00	0.02	0.02	0.00
TRBC	14	16	0.02	0.03	0.04	0.04	0.02
TRBC	14	17	0.00	0.09	0.03	0.02	0.02
TRBC	15	17	0.06	0.09	0.04	0.00	0.03
TRBC	15	16	0.00	0.02	0.05	0.00	0.03

TRBC	15	14	0.00	0.05	0.02	0.00	0.00
TRBC	15	11	0.09	0.03	0.00	0.00	0.00
TRBC	15	9	0.03	0.06	0.09	0.00	0.00
TRBC	15	7	0.13	0.10	0.16	0.00	0.00
TRBC	15	6	0.03	0.04	0.14	0.00	0.10
TRBC	15	5	0.00	0.05	0.09	0.00	0.10
TRBC	15	3	0.09	0.08	0.04	0.00	0.00
TRBC	15	2	0.09	0.01	0.11	0.00	0.10
TRBC	16	19	0.00	0.00	0.00	0.00	0.00
TRBC	16	17	0.00	0.29	0.08	0.00	0.00
TRBC	16	16	0.00	0.00	0.00	0.10	0.00
TRBC	16	14	0.00	0.00	0.00	0.00	0.00
TRBC	16	11	0.24	0.00	0.00	0.05	0.07
TRBC	16	6	0.00	0.00	0.15	0.05	0.07
TRBC	16	3	0.00	0.00	0.00	0.00	0.00
TRBC	16	2	0.00	0.00	0.00	0.05	0.00
TRBC	17	3	0.00	0.04	0.00	0.00	0.03
TRBC	17	5	0.03	0.00	0.02	0.00	0.02
TRBC	17	6	0.00	0.02	0.00	0.00	0.10
TRBC	17	9	0.03	0.06	0.00	0.03	0.02
TRBC	17	11	0.01	0.02	0.00	0.00	0.00
TRBC	17	14	0.08	0.00	0.07	0.05	0.03
TRBC	17	16	0.04	0.13	0.05	0.00	0.00
TRBC	17	17	0.07	0.00	0.02	0.00	0.02
TRBC	17	19	0.01	0.02	0.02	0.00	0.00
TRBC	18	19	0.37	0.47	0.82	0.50	0.63
TRBC	18	17	0.11	0.10	0.13	0.08	0.11
TRBC	18	16	0.04	0.07	0.06	0.00	0.07
TRBC	18	14	0.00	0.09	0.12	0.08	0.03
TRBC	18	11	0.13	0.12	0.17	0.11	0.12
TRBC	18	6	0.13	0.12	0.11	0.11	0.12
TRBC	18	4	0.03	0.14	0.09	0.18	0.05
TRBC	18	3	0.08	0.14	0.05	0.07	0.05
TRBC	18	1	0.03	0.07	0.08	0.07	0.03
TRBC	19	1	0.00	0.00	0.00	0.00	0.00
TRBC	19	2	0.00	0.00	0.00	0.00	0.00
TRBC	19	3	0.00	0.79	0.00	0.00	0.00
TRBC	19	5	0.00	0.00	0.00	0.00	0.00
TRBC	19	6	0.00	0.00	0.00	0.00	0.00
TRBC	19	7	0.00	0.84	0.00	0.00	0.00
TRBC	19	9	0.00	0.00	0.00	0.00	0.00
TRBC	19	11	0.00	0.00	0.00	0.00	0.00
TRBC	19	14	0.00	0.00	0.00	0.00	0.00
TRBC	19	16	0.00	0.00	0.00	0.00	0.00
TRBC	19	17	0.00	0.89	0.00	0.00	0.00
TRBC	19	19	0.00	0.00	0.00	0.00	0.00
TRBC	20	3	0.01	0.02	0.01	0.02	0.01

TRBC	20	9	0.01	0.01	0.01	0.01	0.01
TRBC	20	11	0.01	0.01	0.03	0.02	0.02
TRBC	20	14	0.01	0.01	0.01	0.01	0.01
TRBC	20	16	0.03	0.02	0.02	0.02	0.02
TRBC	20	17	0.02	0.03	0.02	0.03	0.03
TRBC	20	19	0.02	0.01	0.01	0.01	0.02

B)

Gene	OT	C position	% base conversion C>T				
			UTD	3CAR BE	7CAR BE	3CAR SP	7CAR SP
CD7	1	16	0.00	0.41	0.00	0.00	0.17
CD7	1	13	0.00	0.01	0.00	0.11	0.00
CD7	1	12	0.00	0.03	0.10	0.00	0.11
CD7	1	8	0.00	4.28	4.44	0.12	0.01
CD7	1	7	0.00	4.37	2.62	0.00	0.03
CD7	1	4	0.00	6.84	7.63	0.00	0.03
CD7	1	3	0.16	8.48	10.06	0.11	0.02
CD7	1	1	0.00	2.60	3.49	0.00	0.00
CD7	2	1	1.53	0.00	0.38	0.00	0.92
CD7	2	3	0.81	0.00	0.20	0.00	0.61
CD7	2	4	1.61	0.00	0.00	0.00	1.41
CD7	2	7	0.26	0.00	0.10	0.11	0.40
CD7	2	12	0.03	0.00	0.20	0.38	0.03
CD7	2	13	0.00	0.00	0.39	0.11	0.00
CD7	2	16	0.05	0.00	0.80	0.78	0.09
CD7	2	18	0.00	0.14	0.39	0.66	0.00
CD7	3	18	0.00	0.00	0.00	0.04	0.00
CD7	3	16	0.00	0.00	0.00	0.08	0.09
CD7	3	13	0.00	0.00	0.00	0.00	0.30
CD7	3	12	0.00	0.00	0.00	0.00	0.10
CD7	3	8	0.21	0.00	0.09	0.05	0.11
CD7	3	7	0.21	1.96	0.00	0.00	0.33
CD7	3	4	0.11	0.00	0.00	0.10	0.00
CD7	3	3	0.00	0.00	0.10	0.05	0.23
CD7	3	2	0.00	0.00	0.00	0.05	0.11
CD7	3	1	0.00	0.00	0.00	0.00	0.00
CD7	4	18	0.00	0.04	0.00	0.18	0.00
CD7	4	16	0.00	0.00	0.03	0.00	0.00
CD7	4	13	0.00	0.00	0.06	0.00	0.12
CD7	4	12	0.00	0.00	0.03	0.00	0.00
CD7	4	8	0.00	0.00	0.03	0.07	0.05
CD7	4	7	0.00	0.11	0.00	0.07	0.14
CD7	4	4	0.00	0.00	0.00	0.00	0.00
CD7	4	3	0.00	0.05	0.00	0.00	0.00
CD7	4	2	0.00	0.05	0.00	0.08	0.05
CD7	4	1	0.00	0.00	0.00	0.00	0.10

CD7	5	18	0.00	0.09	0.00	0.00	0.07
CD7	5	16	0.00	0.00	0.00	0.00	0.00
CD7	5	13	0.00	0.00	0.00	0.00	0.00
CD7	5	8	0.14	0.00	0.08	0.00	0.00
CD7	5	7	0.00	0.08	0.00	0.00	0.00
CD7	5	4	0.00	0.00	0.00	0.00	0.00
CD7	5	3	0.00	0.00	0.00	0.00	0.00
CD7	5	1	0.00	0.00	0.00	0.00	0.00
CD7	6	1	0.02	0.04	0.05	0.00	0.06
CD7	6	3	0.04	0.09	0.01	0.06	0.01
CD7	6	4	0.01	0.00	0.09	0.10	0.04
CD7	6	7	0.00	0.02	0.01	0.00	0.01
CD7	6	12	0.03	0.01	0.04	0.02	0.02
CD7	6	13	0.04	0.00	0.01	0.02	0.01
CD7	6	16	0.05	0.01	0.01	0.02	0.01
CD7	6	18	0.04	0.02	0.07	0.04	0.07
CD7	7	1	0.02	0.03	0.06	0.04	0.04
CD7	7	4	0.07	0.09	0.06	0.05	0.04
CD7	7	5	0.11	0.09	0.08	0.02	0.04
CD7	7	7	0.04	1.88	1.63	0.02	0.04
CD7	7	12	0.09	0.03	0.02	0.04	0.03
CD7	7	13	0.00	0.04	0.05	0.05	0.05
CD7	7	16	0.02	0.07	0.06	0.03	0.04
CD7	7	18	0.15	0.09	0.06	0.05	0.07
CD7	8	1	0.02	0.04	0.03	0.01	0.04
CD7	8	4	0.05	0.03	0.04	0.02	0.04
CD7	8	7	0.04	0.03	0.03	0.01	0.02
CD7	8	8	0.14	0.06	0.07	0.06	0.12
CD7	8	12	0.03	0.01	0.03	0.02	0.04
CD7	8	13	0.03	0.03	0.04	0.05	0.04
CD7	8	16	0.05	0.04	0.04	0.04	0.07
CD7	8	18	0.01	0.01	0.02	0.01	0.02
CD7	9	18	0.01	0.01	0.02	0.02	0.02
CD7	9	16	0.02	0.02	0.02	0.01	0.03
CD7	9	13	0.03	0.05	0.04	0.03	0.04
CD7	9	12	0.04	0.01	0.02	0.02	0.02
CD7	9	7	0.04	0.08	0.07	0.04	0.05
CD7	9	4	0.03	0.02	0.04	0.03	0.01
CD7	9	3	0.03	0.03	0.04	0.03	0.03
CD7	9	2	0.02	0.03	0.03	0.04	0.03
CD7	10	1	0.03	0.03	0.02	0.03	0.07
CD7	10	3	0.02	0.02	0.05	0.02	0.03
CD7	10	4	0.02	0.01	0.07	0.02	0.03
CD7	10	7	0.04	0.02	0.11	0.04	0.04
CD7	10	8	0.05	0.06	0.09	0.03	0.08
CD7	10	12	0.04	0.02	0.04	0.04	0.04
CD7	10	13	0.04	0.03	0.08	0.03	0.04

CD7	10	16	0.04	0.02	0.04	0.02	0.01
CD7	10	18	0.03	0.02	0.01	0.03	0.02
CD7	11	18	0.07	0.00	0.12	0.00	0.00
CD7	11	16	0.07	0.00	0.00	0.12	0.00
CD7	11	13	0.07	0.11	0.00	0.00	0.07
CD7	11	12	0.07	0.00	0.00	0.00	0.07
CD7	11	11	0.07	0.11	0.00	0.18	0.00
CD7	11	8	0.07	0.11	0.23	0.00	0.00
CD7	11	7	0.00	0.22	0.47	0.00	0.00
CD7	11	4	0.00	0.11	0.12	0.06	0.07
CD7	11	1	0.07	0.06	0.06	0.00	0.00
CD7	12	1	0.00	0.02	0.02	0.00	0.03
CD7	12	3	0.05	0.02	0.07	0.04	0.04
CD7	12	4	0.00	0.01	0.03	0.00	0.03
CD7	12	7	0.00	0.03	0.04	0.03	0.01
CD7	12	8	0.15	0.06	0.09	0.07	0.02
CD7	12	11	0.00	0.03	0.07	0.00	0.03
CD7	12	12	0.05	0.04	0.08	0.00	0.04
CD7	12	13	0.05	0.03	0.04	0.03	0.02
CD7	12	16	0.00	0.03	0.04	0.03	0.02
CD7	12	18	0.05	0.05	0.12	0.10	0.06
CD7	13	1	0.03	0.09	0.04	0.03	0.03
CD7	13	3	0.05	0.05	0.04	0.03	0.03
CD7	13	4	0.02	0.00	0.06	0.04	0.07
CD7	13	8	0.03	0.00	0.05	0.04	0.06
CD7	13	12	0.05	0.05	0.06	0.04	0.07
CD7	13	13	0.01	0.05	0.07	0.03	0.03
CD7	13	16	0.07	0.00	0.08	0.07	0.08
CD7	13	18	0.05	0.00	0.05	0.05	0.07
CD7	14	1	0.04	0.02	0.05	0.07	0.04
CD7	14	2	0.07	0.06	0.04	0.05	0.05
CD7	14	4	0.01	0.00	0.01	0.01	0.05
CD7	14	7	0.03	0.04	0.03	0.04	0.04
CD7	14	8	0.02	0.04	0.04	0.08	0.04
CD7	14	13	0.04	0.02	0.03	0.04	0.03
CD7	14	16	0.02	0.00	0.04	0.01	0.03
CD7	14	18	0.04	0.02	0.04	0.03	0.04
CD7	15	18	0.34	0.08	0.37	0.38	0.33
CD7	15	16	0.42	0.24	0.48	0.29	0.34
CD7	15	13	0.19	0.00	0.33	0.33	0.18
CD7	15	12	0.19	0.33	0.08	0.21	0.13
CD7	15	8	0.35	0.09	0.34	0.37	0.24
CD7	15	4	0.12	0.00	0.10	0.15	0.08
CD7	15	3	0.23	0.17	0.26	0.18	0.13
CD7	16	3	0.00	0.00	0.00	0.00	0.00
CD7	16	4	0.36	0.00	0.31	0.00	0.00
CD7	16	8	0.68	0.00	0.00	0.00	0.00

CD7	16	12	0.00	0.00	0.00	0.00	0.00
CD7	16	13	0.00	0.00	0.31	0.00	0.00
CD7	16	16	0.00	0.00	0.00	0.00	0.00
CD7	16	18	0.00	0.00	0.00	0.00	0.00
CD7	17	1	0.06	0.06	0.00	0.00	0.11
CD7	17	4	0.06	0.06	0.05	0.07	0.02
CD7	17	5	0.00	0.06	0.11	0.00	0.02
CD7	17	7	0.05	1.54	1.10	0.06	0.04
CD7	17	8	0.00	1.03	0.71	0.13	0.06
CD7	17	13	0.00	0.00	0.02	0.06	0.02
CD7	17	16	0.11	0.06	0.10	0.07	0.10
CD7	17	18	0.00	0.06	0.02	0.13	0.00
CD7	18	16	0.05	0.15	0.03	0.07	0.09
CD7	18	13	0.04	0.00	0.05	0.01	0.03
CD7	18	12	0.02	0.02	0.00	0.03	0.01
CD7	18	11	0.04	0.11	0.06	0.05	0.02
CD7	18	8	0.02	0.11	0.01	0.00	0.04
CD7	18	7	0.05	0.04	0.04	0.05	0.10
CD7	18	4	0.06	0.02	0.01	0.02	0.02
CD7	18	3	0.11	0.02	0.06	0.07	0.04
CD7	18	1	0.04	0.08	0.03	0.04	0.05
CD7	19	2	0.03	0.01	0.03	0.02	0.04
CD7	19	3	0.01	0.00	0.03	0.01	0.03
CD7	19	4	0.02	0.03	0.00	0.02	0.05
CD7	19	7	0.04	0.05	0.03	0.03	0.01
CD7	19	8	0.01	0.06	0.00	0.01	0.03
CD7	19	12	0.05	0.06	0.02	0.06	0.03
CD7	19	13	0.04	0.08	0.01	0.03	0.03
CD7	19	16	0.01	0.02	0.03	0.02	0.06
CD7	19	18	0.01	0.05	0.01	0.02	0.02
CD7	20	2	0.06	0.30	0.37	0.05	0.07
CD7	20	3	0.05	0.19	0.19	0.04	0.04
CD7	20	4	0.07	0.46	0.41	0.06	0.05
CD7	20	6	0.04	3.15	4.08	0.03	0.04
CD7	20	7	0.02	2.96	3.77	0.02	0.04
CD7	20	8	0.03	2.71	3.67	0.02	0.03
CD7	20	12	0.03	0.04	0.03	0.05	0.05
CD7	20	13	0.03	0.03	0.05	0.02	0.05
CD7	20	16	0.03	0.10	0.19	0.03	0.04
CD7	20	18	0.05	0.04	0.05	0.05	0.04

Supplementary Table 3

Translocation Number	Probe Sequence
1, 2	ACAACCTGACTATCACCATGCACCGC
3	TACGGCTCCGGCACCCTGGT
4	CTGCCAGGCCATCACGGAGGTCAAT
albumin	CCTGTCATGCCCACACAAATCTCTCC
**Translocation Number**	**Primer Sequence**
1 Fwd	AGGTCGCTGTGTTTGAGC
1 Rev	AGGTCGCTGTGTTTGAGC
2 Fwd	ACTACGGACAGACGGTTC
2 Rev	TATCTGGAGTCATTGAGGGC
3 Fwd	ATCTGGAGTCATTGAGGGCG
3 Rev	TGGGCACATTCCCTACCT
4 Fwd	AGGTCGCTGTGTTTGAGC
4 Rev	AGGTCGCTGTGTTTGAGC
Albumin Fwd	GCTGCTATCTCTTGTGGGCTGT
Albumin Rev	ACTCATGGGAGCTGCTGGTTC

Primer and probe sets for translocation detection using ddPCR.

Supplementary Table S4. Primer sequences for **A)** TRBC and **B)** CD7 off-targets. Primers are given in the 5'-3' orientation. Fwd: forward, Rev: reverse.

A)

Gene Target	Notes	Sequence
TRBC-01	Fwd primer	GAGGCAGGTCTTTCACATTCA
TRBC-01	Rev primer	CAAAACGGAAGGAGGCATGT
TRBC-02	Fwd primer	AATCTTCACAGCCCCTCCTC
TRBC-02	Rev primer	AGTGCGGCCTCAGGTAGC
TRBC-03	Fwd primer	GAGAGTGAACGAGGGTGTGG
TRBC-03	Rev primer	CCACCTTCACCCCTCGAG
TRBC-04	Fwd primer	GTGGTAAGTGTGAGCATTGCAG
TRBC-04	Rev primer	TCAGTCCATACTTTCCCCGC
TRBC-05	Fwd primer	GATACCTGCTGTCCCCTCTC
TRBC-05	Rev primer	CAGAGGTAGATTGCTATGGACCT
TRBC-06	Fwd primer	CTTATCCCAGCAGCACTTGG
TRBC-06	Rev primer	TCTCTTCCTCCCACAACACC
TRBC-07	Fwd primer	AAAATATGGCCATGCATTTT
TRBC-07	Rev primer	AAAGACACATTCATTTTCCC
TRBC-08	Fwd primer	AGAAAGTTGCTCAATCAATG
TRBC-08	Rev primer	TAGAGGGAGAAAGAAACAAC
TRBC-09	Fwd primer	TCTATGCGAGTTTATCTGTC
TRBC-09	Rev primer	CAGCAGGGATCTAAAGAG
TRBC-10	Fwd primer	TAGATGTTTGGCCTGTTTAA
TRBC-10	Rev primer	TGACTTTTGTTTGTTTACCC
TRBC-11	Fwd primer	AAAAGTTTCACCTCTCACAA
TRBC-11	Rev primer	ACAAATTCACATGGCTTTC
TRBC-12	Fwd primer	CTCTCCAAATCCTAGACCT
TRBC-12	Rev primer	CTCCTTTTGCAGAGTTTG
TRBC-13	Fwd primer	CCTAGTGTCATCCTGGAA
TRBC-13	Rev primer	CTCCCATTTTCTCTGCTG
TRBC-14	Fwd primer	CACTTTACTGTAGCCTGG
TRBC-14	Rev primer	TGTGGAGATTGTTAACTAGG
TRBC-15	Fwd primer	CACACCATTCTCATCTTCTA
TRBC-15	Rev primer	AAAGAAGACAAACAACCCA
TRBC-16	Fwd primer	TTTGCAGATGAGGACCTG
TRBC-16	Rev primer	CAAGGTACTCACCGTGTA
TRBC-17	Fwd primer	CTGTTTGTTGTCCATGGA
TRBC-17	Rev primer	GTCAAAAGACATGGCAAATA
TRBC-18	Fwd primer	GGTCCCTGATTCCTTTAAG
TRBC-18	Rev primer	AGACACACACACATATAGAC
TRBC-19	Fwd primer	GTGTTTCTGTGGTTTCAAG
TRBC-19	Rev primer	GTCTGCTTTGCTGTCTGT
TRBC-20	Fwd primer	ATGTAGACTAGCTGAAGTTG
TRBC-20	Rev primer	GGATTTGGGGTACTCTAAAA

B)

Gene Target	Primer	Sequence
CD7-01	Fwd primer	TGTCATGCTGAGTCCACTGT
CD7-01	Rev primer	GACATGTCTCCCCTCCTGTG
CD7-02	Fwd primer	CAATAGCCACACACACCTGG
CD7-02	Rev primer	ACGCCAAGAGAAGTAAGCCT
CD7-03	Fwd primer	TCTCCTCTTTTCGCCTTGGT
CD7-03	Rev primer	GGGAAACTCGTGTACAAGGC
CD7-04	Fwd primer	TCTCCTCTTTTCGCCTTGGT
CD7-04	Rev primer	GGGAAACTCGTGTACAAGGC
CD7-05	Fwd primer	CATAGGCTCCCGAGGATTGA
CD7-05	Rev primer	GCGTGTGTGTGTGATCTCTG
CD7-06	Fwd primer	CAAATGCAGCAAGAACAGGAAC
CD7-06	Rev primer	GTGGTTTTAGGGTGACTGCG
CD7-07	Fwd primer	AATCCTACGTAAAGTACCAG
CD7-07	Rev primer	GCACACCTGAAAGTTCTAA
CD7-08	Fwd primer	GTGGTGGCTACTATTATCAT
CD7-08	Rev primer	CCATCATACTCTCTCATCAG
CD7-09	Fwd primer	CAATGAACACCAAAAGATGA
CD7-09	Rev primer	ATGATCATTTCTGTGTAGCT
CD7-10	Fwd primer	GCAACCTAGTGTCAATATCT
CD7-10	Rev primer	CCTATCAGCACTTGTATTGA
CD7-11	Fwd primer	TTTAGTAGAGACGGGGTTT
CD7-11	Rev primer	TTGACTCTTCTGTGTGTTTT
CD7-12	Fwd primer	TACCTCTCCTCAATTTCAAG
CD7-12	Rev primer	CTTTCAGGAGATTCTTTACG
CD7-13	Fwd primer	CTTGACTTTCAGAGAAGCTA
CD7-13	Rev primer	ACTCATGCCCCAGTTTAT
CD7-14	Fwd primer	TAAGTAACTGGGAAATTGCA
CD7-14	Rev primer	CACCTGTAACTAAGCCAG
CD7-15	Fwd primer	AAGTACTACTGTTCAGGTTC
CD7-15	Rev primer	GTAGAATCACTTGAACCCA
CD7-16	Fwd primer	TACAAATAGCTACAGGGAAG
CD7-16	Rev primer	CTGTAATCCCAGCTACTTG
CD7-17	Fwd primer	GCTTTTGTATCCAAGTCTTT
CD7-17	Rev primer	AACTTACCTTCTGCCACT
CD7-18	Fwd primer	AGACCCTCACTATTCTGG
CD7-18	Rev primer	ATCCCAGCTTTTACAGTTTA
CD7-19	Fwd primer	TGTATACAAAACTGGGTTCT
CD7-19	Rev primer	CTGGGTGAAGGACATTTC
CD7-20	Fwd primer	TTGCAATTCACGGAAATTTA
CD7-20	Rev primer	TCAAAAGCTTCCTAAGTCTT
